# Plant Microbial Biostimulants as a Promising Tool to Enhance the Productivity and Quality of Carrot Root Crops

**DOI:** 10.3390/microorganisms9091850

**Published:** 2021-08-31

**Authors:** Virgilija Gavelienė, Božena Šocik, Elžbieta Jankovska-Bortkevič, Sigita Jurkonienė

**Affiliations:** Nature Research Centre, Laboratory of Plant Physiology, Akademijos Str. 2, LT-08412 Vilnius, Lithuania; bozena.socik@gamtc.lt (B.Š.); elzbieta.jankovska@gamtc.lt (E.J.-B.)

**Keywords:** antioxidant activity, conventional farm, *Daucus carota*, field probiotic application, monosaccharides, organic farm, plant probiotic microorganisms, vitamin C

## Abstract

The interest in studies focused on applying probiotic microorganisms is increasing due to sustainable agriculture development. In this research, we aimed to evaluate the impact of two commercial plant probiotics—ProbioHumus and NaturGel on carrot growth, yield, and quality in organic and nonorganic production systems. The research was carried out under laboratory and field conditions. Plants were treated with probiotics (2 L/ha) at the nine leaves stage. Biometrical measurements and chemical analyses were performed at a maturation stage. The average weight of carrot roots increased by 17 and 20 g in the test variant with ProbioHumus as compared to the control in the organic and nonorganic farms, respectively. Plant microbial biostimulants ProbioHumus and NaturGel had a positive effect on the quality of carrots from organic and nonorganic farms: applied in couple they promoted the accumulation of monosaccharides, ascorbic acid, carotenoids, phenols, and increased antioxidant activity. Quantitative nitrate analysis regardless of the biostimulant used revealed about twofold lower nitrate content of carrots from organic than nonorganic farms, and probiotics did not show a significant effect on nitrate accumulation. Finally, ProbioHumus and NaturGel were effective at low doses. The use of microbial biostimulants can be recommended as an element of cultivation for creating ecologically friendly technologies.

## 1. Introduction

The abundant use of unbalanced chemical fertilizers leads to food safety and quality decline problems [1,2]. In recent years, researchers focused on applying plant-beneficial microorganisms (probiotics) to partially replace chemical fertilizer use, are increasing due to the requirement of sustainable agriculture development and the European Green Deal initiative. Plant probiotic microorganisms are known as bioprotectants, biocontrollers, biofertilizers, or biostimulants [3,4,5]. Plant biostimulants were defined as any substance or microorganisms supplied to plants primarily with the aim of enhancing the growth and yield of the plants, and increasing crop quality traits, with regard to their nutrients content [4,6,7,8]. It is thought that probiotics could serve as a tool to produce highly functional foods, hence benefiting human health in a dual way, namely the replacement of chemical fertilizers by biofertilizers and the increase in bioactive compounds [1]. Indeed, several studies have demonstrated that probiotics are an alternative that has the potential to minimize the negative influence on vegetables, such as nitrate accumulation by using chemical fertilization [9,10]. Organic farming, which strictly prohibits synthetic fertilizers, provides products endowed with improved nutritional properties [11,12]. Therefore, plant probiotics, applied as biofertilizers could serve as a possible solution to improve the food quality of agricultural crops and vegetables. However, organic farming is always associated with a lower yield of crops and thus a higher cost. Therefore, the use of chemical fertilizers is not able to be eliminated once considerable food production is expected [13,14]. The development of innovating probiotic products is based on microbiology applied to agriculture. The concept of Effective Microorganisms (EM) was developed by Higa and Parr [15]. According to their research, the inoculation of EM cultures to the soil/plant ecosystem can improve the growth, yield, and quality of crops [15]. The supply of biofertilizers has been increasing in recent years, and the use of probiotics on nonorganic and organic farms is expanding every year. Selected plant probiotic microorganisms produced on a commercial scale by companies are tested in the open field for their ability to properly feed the crop and ensure crop quality [5,16]. In Lithuania and Latvia, recently developed microbial biostimulants plant probiotics ProbioHumus and NaturGel were used for agriculture and are available for vegetable and crop production in the biofertilizer market.

Thus, there is a need to study the aspects of the impact of microbial biostimulants on crop growth and development and implement their application to modern agriculture. There are few studies about the effects of plant probiotics on the yield of vegetables, and especially, on the content of their bioactive compounds [5,12,17,18]. The increase in sugar accumulation and yield was observed in sugar beet roots after treatment with microbial biostimulants [19,20]. Additionally, Bona with coworkers [21] revealed that inoculation with the strain Pseudomonas sp. 19Fv1T not only enhanced yield but also positively affected the concentration of ascorbic acid in tomato fruits. A study on vermicompost combined with plant probiotic *Bacillus megatherium* and *B. amyloliquefaciens* [22] showed increased tomato yield and vitamin C contents. According to [23], the highest levels of vitamin C content in tomato fruits were obtained after the inoculation of two bacterial strains *B. amyloliquefaciens* (FZB2 and FZB42) in different treatments. We also found data that the use of biofertilizers in the cultivation of carrots led to an increase in the produced biomass, and modified the chemical composition of roots [2,24,25]. Nevertheless, there is still a lack of knowledge about the effects of probiotics on carrot productivity and yield quality. Carrots are particularly a good source of antioxidants with a 10-fold greater capacity of scavenging free radicals than that of many other vegetables [26]. Ascorbic acid is in part responsible for the antioxidant property of carrots together with a wide variety of phenolics, including hydroxybenzoic and hydroxycinnamic acid derivates and flavonols [27]. Some studies have shown that carrots grown with organic fertilizers have a better sweetness than with chemical fertilizers [28,29]. In addition, organic fertilizers can increase the content of nutrients such as β-carotenes [30]. So far, few studies have been focused on the effect of plant probiotics on carrot crop quality.

Thus, we hypothesized that probiotics could influence the growth, yield, and quality of carrots and could serve as a tool to produce highly functional foods hence benefiting human health in a dual way, namely replacing chemical fertilizers with biostimulants and enhancing the food quality of vegetables. Plant probiotic compositions ProbioHumus produced by Latvian and NaturGel produced by Lithuanian manufacturer were tested in our study. In this research, we aimed to evaluate the impact of two plant commercial probiotics—ProbioHumus and NaturGel on carrots growth, yield, and its quality in organic and conventional production systems.

## 2. Materials and Methods

### 2.1. Plant Material and Treatments

**Figure 1:**
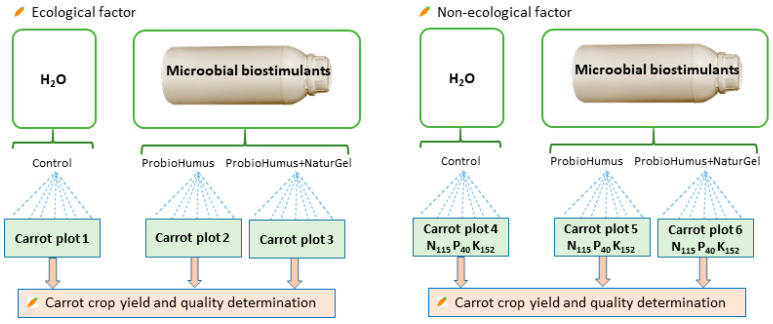
The design of the experiment.

The root crop carrot (*Daucus carrota* L.) cv. ‘Nipomo H’ was grown in controlled laboratory and field conditions. Probiotics ProbioHumus and NaturGel were used as biostimulants to enhance the growth, productivity and quality of carrots. Two factors were used in the study: ecological—in organic plots and nonecological—in nonorganic plots (Figure 1). Entirely probiotic preparations as biostimulants were used in organic plots. The combination of probiotic preparations with application of mineral fertilizers: N_115_, P_40_ and K_152_ kg/ha was used in nonorganic plots.

### 2.2. Probiotics ProbioHumus and NaturGel

The impact of two commercial probiotics was analyzed in the present study. The commercial probiotic preparation ProbioHumus (purchased from Baltic Probiotics, Latvia) is a composition of microorganisms: *Bacillus subtilis*, yeast *Saccharomyces cerevisiae*, lactic acid bacteria *Bifidobacterium animalis*, *B. bifidum*, *B. longum*, *Lactobacillus diacetylactis*, *L. casei*, *L. delbrueckii*, *L. plantarum*, *Lactococcus lactis*, *Streptococcus thermophilus*, phototropic bacteria *Rhodopseudomonas palustris*, and *R. sphaeroides*.

Commercial organic fertilizer (probiotic preparation) NaturGel contains ferments, amino acids, vitamins (B_1_, B_2_, PP, E, A, carotenoids), fulvo and humic acids, carbohydrates, microorganisms from *Azotobacter*, *Bacillus*, *Rhizobium*, *Bradyrhizobium*, *Lactobacillus*, and *Trichoderma* genera, macroelements: nitrogen, phosphorus, and potassium, and microelements: magnesium, iron, manganese, zinc, and copper (purchased from Sadera, Lithuania).

### 2.3. Determination of the Active Probiotic Concentration

To determine the active probiotic concentration, seeds of carrot were sown in 10 × 3 pots with peat substrate in a Climacell plant growing chamber (Medcenter Einrichtangen GmbH) at a constant temperature 24 °C under the illumination of 60 mol m^2^ s^−1^ and photoperiod 16/8 h and at 65% humidity [31]. Each experimental unit consisted of 14 seeds. Pots without biostimulants served as control. Seven treatments of the study of probiotic impact were used: (1) control; (2) ProbioHumus 1 mL/100 mL; (3) ProbioHumus 2 mL/100 mL; (4) ProbioHumus 4 mL/100 mL; (5) NaturGel 1 mL/100 mL; (6) NaturGel 2 mL/100 mL; (7) NaturGel 4 mL/100 mL; 8) ProbioHumus 1 mL/100 mL + NaturGel 1 mL/100 mL; (9) ProbioHumus 2 mL/100 mL + NaturGel 2 mL/100 mL; (10) ProbioHumus 4 mL/100 mL + NaturGel 4 mL/100 mL. Plants were foliar sprayed with water solutions of tested preparations applied in 10 mL solutions for each growing dish at the 4–9 leave stage (BBCH 14–19) [32], control was sprayed with water. Carrots were collected after 72 days of cultivation and morphometric parameters were measured right away.

### 2.4. Small Plot Field Experiments

Small plot trials (1 m^2^) with carrot crops were carried out at the Experimental Field Station of the Nature Research Centre (Lithuania) (54°68′ N 25°26′ E) on light loamy Endocalcari-Epihypogleyic Cambisol in 4-fold repetition completed in randomized blocks. The main agrochemical parameters of the arable soil layer were pH 7.0–7.3, N_min_ 1.5–4.51 kg/ha, P_2_O_5_ 248–250 mg/ha and K_2_O 214.0–214.6 mg/kg. Each plot was foliar sprayed with 100 mL of the most active doses of tested preparations (2 mL/100 mL) at the BBCH 14–19. In the organic plots, entirely (only) probiotic preparations were used, in nonorganic plots, they were used in combination with the application of mineral fertilizers. The morphometric parameters of plant growth at the stage of technical maturity (roots mass, length, and diameter) were estimated in 30 plants of each investigated variant.

### 2.5. Large Plot Field Experiments

Carrot plants were cultivated under field conditions in an organic and nonorganic commercial farm in Pasvalys distr., Lithuania, 56°12′ N 24°25′44 E on typical Endohypogleyic Arenosol. The main agrochemical parameters of the arable soil layer of the organic farm were pH 7.0–7.3, N_min_ 2.3–8.42 kg/ha, P_2_O_5_ 109–166 mg/ha, K_2_O 97.0–177 mg/kg, and of the nonorganic farm—pH 7.0–7.3, N_min_ 3.1–18.2 kg/ha, P_2_O_5_ 248–250 mg/ha and K_2_O 114.0–224.6 mg/kg. Three controlled fields (3 × 1 ha) were arranged in order to test probiotics: (1) control without any treatment, (2) ProbioHumus, and (3) ProbioHumus and NaturGel in couple. Carrots were treated at 9 leaves stage (BBCH 18–19) using 2 L/ha of preparations in organic farms and, using the same volume of preparations combined with application of mineral fertilizers in nonorganic farms. Yield productivity indicators: the mass of roots, their diameter, and length were measured at the stage of technical maturity before harvesting. A randomly selected sample of the yield of 20 kg was taken from each test variant.

### 2.6. Carrot Crop Yield Quality Determination

#### 2.6.1. Sample Preparation

The nonedible parts were removed, and the samples of carrot roots were subjected to cutting and homogenization using a homogenizer. The homogenization of 5 kg carrots and random selection of samples from the mass was used to avoid the uncertainty due to sample nonhomogeneity. Then, the one part of homogenized samples was immediately stored at −80 °C before the analysis. Another part of the samples was dried in a drying oven for 24 h at 105 °C and homogenized until powder for monosaccharide content analysis.

#### 2.6.2. Quantitative Analysis of Monosaccharides

The content of monosaccharides was determined in dray carrot roots. The plant material was homogenized in 80% ethanol. After centrifugation at 3000× *g* rpm (MPW-351 R, Poland) amount of carbohydrates was detected by orcinol method [33]. The degree of monosaccharides absorption was determined at wavelength 452 nm with spectrophotometer Specord 210 Plus (Analytic, Jena, Germany) and calculated by standard calibration curve formed on the basis glucose uptake (0.1 mg/100 mL).

#### 2.6.3. Determination of Total Carotenoids

The total carotenoid content analysis was performed in N,N–dimethylformamide extracts. Attempts were performed using 3 replicates of fresh carrot tissues from each variant of three biological repeats. Extraction lasted for 4 days at 4 °C. Absorption of the extract was measured at 480 nm, 647 nm, and 664 nm spectrophotometrically. The amount of carotenoids was calculated according to the formula [34]:

C = [1000 × A_480_ − 0.89 × (11.65 × A_664_ − 2.69 × A_647_) − 52.0 × 2 (20.8 × 1A_647_ − 4.53 × A_664_)]/245 (1)

A—absorption.

The amount of carotenoids per unit of fresh weight per unit was calculated by the following formula:

P = ((C × V) × dilutions times)/M × 1000
(2)

where P—pigments content in mg/g of fresh mass, C—concentration in mg/L, V—pigment extracts volume, M –fresh mass in grams.

#### 2.6.4. Determination of Ascorbic Acid

Amount of ascorbic acid was determined according to the HPTLC method reported by Chakraborthy with minor modifications [35]. Standard solutions of ascorbic acid were prepared by dissolving it in absolute ethanol (99.8%). The standard solution and sample extracts were transferred to a 20 cm × 10 cm glass silica gel chromatography plate using an TLC 4 automated sampler (Camag, Muttenz, Switzerland). For analysis, 5 g of the frozen carrots were homogenized with 50% methanol, and the solution was centrifuged at 4 °C for 15 min at 3000× *g* rpm. The extracts were stored at 4 °C in the dark for 16 h until evaluation. Samples were sprayed with a 25 µL dosing syringe (Hamilton, OH, USA) as a 6 mm bands. The automatic developing chamber ADC 2 provided a temperature of 24 °C and relative humidity conditions of 33 %. The mixture of the ethanol (96%)—glacial acetic acid in a volume composition of 9.5:0.5 (*v*/*v*) was used as the mobile phase. A total of 35 mL of the mobile was used in all cases. A twin-trough chamber was used for the chromatogram development. When the sample components have been separated, the air-dried plate was scanned at 256 nm using a visualizer TLC 2 (Camag, Muttenz, Switzerland). Spectro-densitometric measurements were conducted by a scanner TLC 4 (Camag, Muttenz, Switzerland) operated in the absorbance mode and controlled by the winCATS 1.4.2 software. The obtained data have been derived from the results of sample peaks, analogous to ascorbic acid in the calibration curve. All reagents were of analytical HPTLC grade Merck (Germany) and standards were from Sigma Chemical Co. (St. Louis, MO, USA).

#### 2.6.5. Determination of Total Phenols

The samples of frozen carrots were extracted by 90% aqueous methanol acidified with 0.1 N hydrochloric acid (Fluka) with a ratio of material to medium 1:10 in a porcelain grinder and stirring with a magnetic stirrer for 30 min. The sealed extract was stirred for 16 h in the dark at 4 °C with a magnetic stirrer, and then the precipitate was removed with a water vacuum pump using a 0.2 μm membrane filter (Whatman). Phenolic compounds were determined spectrophotometrically using 10% Folin–Ciocalteu reagent (Sigma-Aldrich, St. Louis, MO, USA) diluted with distilled water and 7.5% Na_2_CO_3_ aqueous solution. For a blank sample a 50% methanol was used. Briefly, 5 mL of 10% Folin–Ciocalteu solution and 4 mL of 7.5% of Na_2_CO_3_ were added to 1 mL of methanol extract of carrot prepared before. Folin–Ciocalteu was added before Na_2_CO_3_, because of avoiding phenol oxidation and sediment formation. The test tubes were shaken and left for 30 min covered in room temperature. After that, the samples were measured spectrophotometrically at 760 nm against the blank sample [36,37].

#### 2.6.6. Evaluation of Antioxidant Activity

The free radical scavenging activity of carrot extracts was measured using DPPH radical coupling method [38]. The frozen carrots 5 g were homogenized in 90% aqueous methanol acidified with 0.1 N hydrochloric acid and stirred with a magnetic stirrer for 30 min, a ratio of material to medium 1:10. The precipitate was removed with a water vacuum pump using a 0.2 μm membrane filter. A freshly prepared 6.5 × 10^−5^ M DPPH (Fluka) methanol (Rotisolv) solution was stirred with a magnetic stirrer for 3 h at 4 °C in the dark. The vegetable extracts were mixed with prepared DPPH solution in ratio 1:20. A control solution based on methanol was prepared accordingly. The solutions were incubated for 30 min at 25 °C in the dark. The decrease of DPPH absorbance was measured with a spectrophotometer at 515 nm. Gallic acid (Sigma-Aldrich, Saint Louis, MO, USA) was used as a standard (R2 = 0.96) and DPPH radical scavenging activity (%) was calculated as (A_c_ − A_s_) × 100/A_c_, where A_c_ is the absorbance of the control (DPPH solution without fruit filtrate) and A_s_ is the absorbance of the sample.

#### 2.6.7. Determination of Nitrate Concentration

The quantification of nitrates was determined in frozen carrot root material using the spectrophotometric method [39]. Samples (5 g of the carrot roots) were extracted by 60 mL hot water (50–60 °C) shaking for 30 min, then clarified using Carrez solution (Sigma-Aldrich) and centrifuged for 10 min at 4000× *g* rpm. Nitrate ions were reduced to nitrite in the presence of zinc powder (Zn) (Sigma-Aldrich). The total nitrite (originally presented in sample plus reduced nitrate) was determined by diazotizing with sulfanilamide and coupling with N-(1-naphthyl)-ethylenediamine dihydrochloride to form an azo dye. The nitrite presented in the sample was determined by measuring without the reduction step. The nitrate was calculated as the difference between the total nitrite content after reduction and the initial nitrite concentration. Color reaction was measured at 540 nm. Nitrate content (mg kg^−1^) was calculated from a calibration curve and expressed on a plant fresh mass basis.

### 2.7. Statistical Analysis

The data were subjected to the analysis of variance (ANOVA). The comparisons for mean values were performed by the Tukey HSD post hoc test. The differences with *p* values of <0.05 were considered to be significant. Different lowercase letters indicate statistically significant difference (*p* < 0.05). Error bars represent the standard deviation of the mean.

## 3. Results

### 3.1. Morphometric Parameters of Carrots under Controlled Conditions

**Figure 2:**
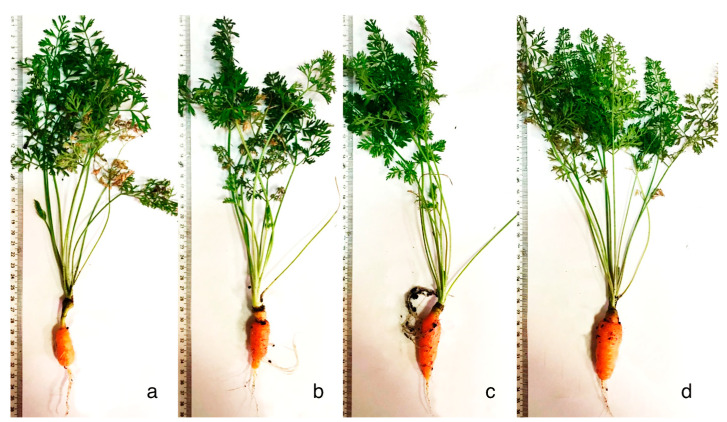
Impact of ProbioHumus and NaturGel on morphometric parameters of carrot cultivated under controlled laboratory conditions. (**a**) Control without treatment; (**b**) ProbioHumus 2 mL/100 mL; (**c**) NaturGel 2 mL/100 mL; (**d**) ProbioHumus + NaturGel 2 + 2 mL/100 mL.

The selection of probiotic concentrations to improve the growth of carrots under laboratory conditions showed that 2 mL/100 mL is more appropriate (Table 1). Final mean weight was observed to be highest in carrots treated with ProbioHumus and ProbioHumus + NaturGel. The length of carrot root almost doubled after treatment with ProbioHumus and ProbioHumus + NaturGel, increasing reach by 80% and 98%, respectively (Table 1, Figure 2). Treatment with probiotic compositions in couple showed the highest vegetable morphometric results compared with control treatment.

### 3.2. Morphometric Parameters of Carrots from Small Plots

Biometric measurements of carrots performed on organic and nonorganic plots showed that the highest average weight of carrots obtained in nonorganic plots in the test variant with ProbioHumus and ProbioHumus + NaturGel—root weight increased by 8–9% as compared to the control. In organic plots, the positive effect was reached in test variant ProbioHumus + NaturGel. From the results presented in Table 2, it is obvious that a corresponding increase in carrot length and diameter resulted in a higher carrot weight. Both probiotic preparations promoted dry matter accumulation (Table 2).

### 3.3. Morphometric Parameters of Carrots from Large Plots

Final mean weight was observed to be highest in carrots treated with ProbioHumus both in organic and in nonorganic farms (Table 3). Moreover, the highest diameter and weight of the root crop was recorded in nonorganic experiment with ProbioHumus treatment. The greatest increase of carrot average length was recorded in crops treated with ProbioHumus + Naturgel in nonorganic farms (24% as compared to control). Measurements of dry matter accumulation in carrots showed that highest dry matter mean value was detected in carrot root samples taken from the organic farm test variant—ProbioHumus. (Table 3).

### 3.4. Monosaccharide Content

Quantitative analysis of monosaccharides in carrot roots showed that tested preparations increased formation of sugars in carrot tissues at least by 18% compared to the control. The highest monosaccharide content was found in carrots grown on an organic farm and exposed to ProbioHumus in combination with NaturGel (Table 4).

### 3.5. Total Carotenoid Content

**Figure 3:**
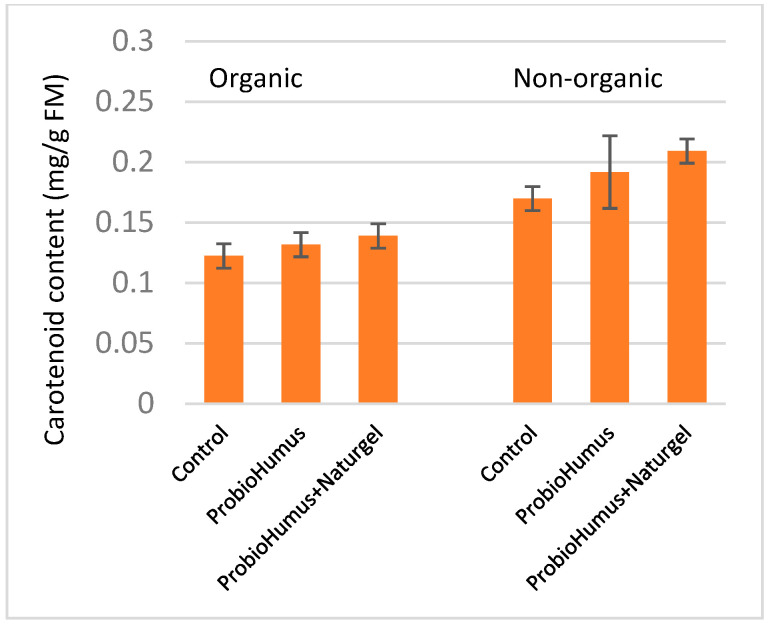
Total carotenoid contents in carrots treated with ProbioHumus and NaturGel. Values reported are mean of three experimental repeats with standard deviation.

Evaluation of carotenoid accumulation in carrot roots showed that the highest levels of these pigments (0.22 mg·g^−1^ FM) were detected in carrots grown on nonorganic farms in couple treated with ProbioHumus and NaturGel. In the organic farm, the tested preparations did not show a significant effect on the accumulation of carotenoids in carrot roots as compared to the control (Figure 3).

### 3.6. Ascorbic Acid Content

**Figure 4:**
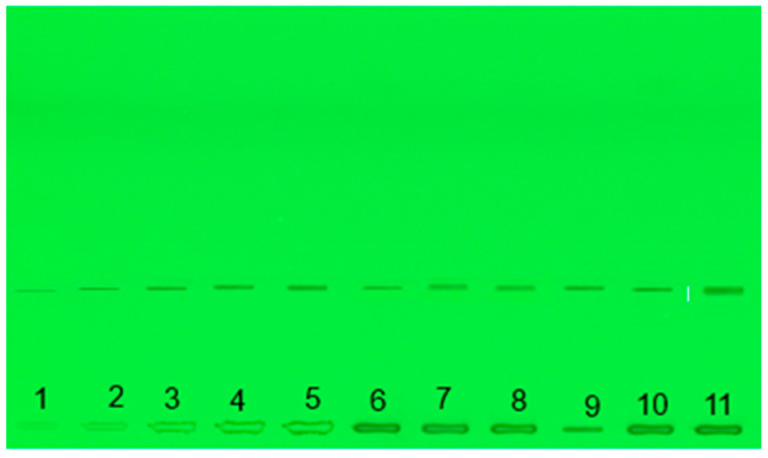
Photograph of HPTLC plate showing the presence of ascorbic acid in *D. carrota* roots at 254 nm. (**1**–**5**) ascorbic acid standards, (**6**–**8**) carrots from nonorganic farm, (**6**) nontreated control, (**7**) ProbioHumus, (**8**) ProbioHumus + NaturGel, (**9**–**11**) carrots from organic farm, (**9**) control, (**10**) ProbioHumus, (**11**) ProbioHumus + NaturGel.

HPTLC analysis of ascorbic acid content showed a 15% and 10% increase of it with the use of ProbioHumus and ProbioHumus + NaturGel, respectively, in carrots from organic farms (Table 5). The highest amount of ascorbic acid was accumulated in nonorganically grown carrots treated with ProbioHumus and NaturGel in a couple. Meanwhile, in carrots grown on organic farms, a significant increase in ascorbic acid content is observed after exposure to ProbioHumus (Table 5, Figure 4).

### 3.7. Total Phenolic Content

Spectrophotometric analysis of phenol concentration indicated that ProbioHumus + NaturGel increased the total phenol accumulation by 10% in carrots from an organic farm. Meanwhile, in carrots grown in nonorganic farm, the content of phenolic compounds rose to 15% after exposure to ProbioHumus (Table 6).

### 3.8. Antioxidant Activity

**Figure 5:**
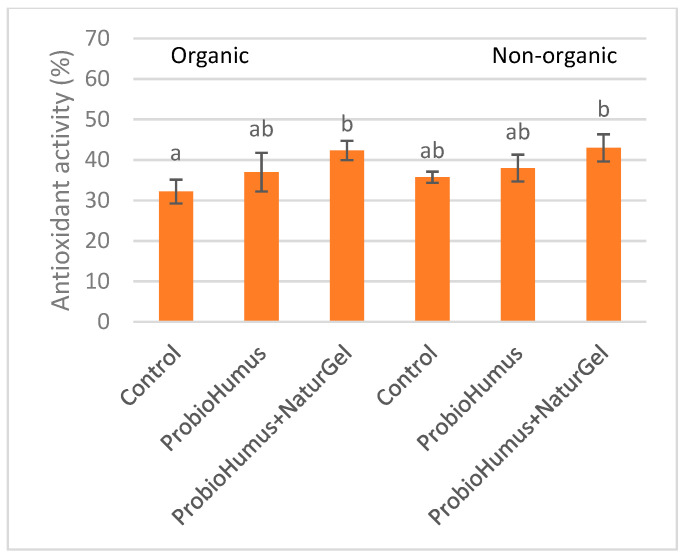
Effect of ProbioHumus and NaturGel on antioxidant activity of carrot roots. Values reported are mean of three experimental repeats with standard deviation. Means with different letters are significantly different (*p* < 0.05).

To evaluate whether ProbioHumus and NaturGel affected the antioxidant activity of carrot roots, we estimated extracts of fresh mass by DPPH assay. The results of the assay demonstrated that ProbioHumus and NaturGel applied in couple increased antioxidant activity of carrots by 10–20% as compared to untreated ones (Figure 5). Meanwhile, ProbioHumus showed better results than control but did not have a statistically significant effect on the antioxidant activity of carrots.

### 3.9. Nitrate Content

**Figure 6:**
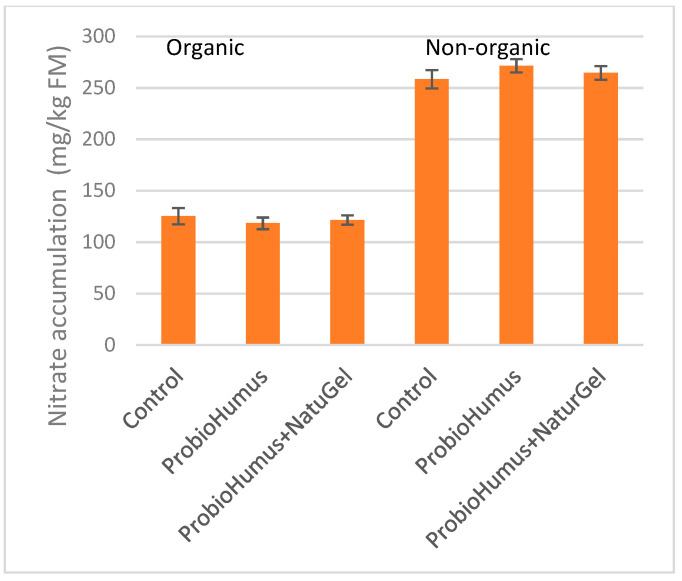
Effect of ProbioHumus and NaturGel on nitrate accumulation in carrot roots. Values reported are mean of three experimental repeats with standard deviation.

Quantitative nitrate analysis showed that organic carrots contain ~2 times fewer nitrates than nonorganic ones. The accumulation of 270 mg/kg of nitrates was exhibited in carrot roots from nonorganic farm. Both probiotic preparations did not show a significant effect on nitrate accumulation (Figure 6).

## 4. Discussion

Carrots are a multinutritional food source. They are an important root vegetable, rich in natural bioactive compounds, which are recognized for their nutraceutical effects and health benefits. Agriculture producers, pushed by the need for high productivity, have stimulated the intensive use of pesticides and fertilizers. Worldwide agricultural practice is moving to a more sustainable and environment-friendly approach due to the increasing demand for safe food and awareness of the environmental and human health damage induced by overuse of pesticides and fertilizers [40]. In addition, the dependence of crop yields on the improvement of agricultural methods and technologies (for example, cultivation, fertilization, irrigation, etc.) is limited, since they do not allow the full use of the biological potential of the crop. There are few studies on the effects of plant probiotics on the yield of functional vegetables and fruits [41,42,43]. The use of biological agents, i.e., probiotic microorganisms can be the potential alternative for chemical fertilizers in crop production and can help to avoid harmful impact on the quality of vegetables and fruits [44,45,46]. Microbial and nonmicrobial plant biostimulants are usually used for open field and greenhouse crops including fruit trees, berry crops, grapevines, vegetables, ornamentals, cereals, and turfs [44,47]. Higa and Parr [15] pointed possibility of using probiotic compositions to avoid chemistry that is too hazardous to the environment. We investigated the efficacy of selected plant probiotic microorganisms labelled as ProbioHumus and NaturGel produced on a commercial scale by Latvian and Lithuanian companies, for the growth of carrot roots under laboratory and natural conditions. We determined that probiotics at 2 mL/100 mL concentrations had biostimulatory properties for carrot growth and development under laboratory and in small plot experiments.

The idea that probiotics are a reliable alternative to the use of chemical fertilizers has led to the need to find out the effectiveness of probiotics when used in combination with mineral fertilizers. Bearing this in mind, we conducted our research under natural conditions not only on organic but also on nonorganic farms. Organic production is one of the fastest-growing food sectors in the world [48,49], though the average yield in the production of organic vegetables still is 33% lower than in nonorganic production. Our study of probiotics in selected concentrations showed enhanced growth and productivity elements formation in organic and nonorganic small and large plots. The highest average carrot weight was obtained in the test variant with ProbioHumus in organic and nonorganic farms (17 and 23% higher compared with control). The treatment of ProbioHumus + NaturGel in couple positively affected the root size, but only in the nonorganic farm. It is thought that microbial biostimulants could be particularly suitable for improving not only the yield but also the quality of root crops. However, we find absolutely no data on the effects of microbial biostimulants on carrot growth and yield quality. Several publications indicate that the use of nonmicrobial biostimulants of plant origin for carrot growth increases biomass and modifies the chemical composition of the roots [2,24,50]. For example, seaweed extracts are considered an important category of nonmicrobial plant biostimulants due to their use on crops under both conventional and organic farming systems. They enhance crop productivity and reduce the use of conventional synthetic fertilizers [51,52]. The study of Italian researchers indicated that microalgae extracts perform biostimulant effects on the expression of root traits related to nutrient acquisition in sugar beet improving plant growth and vigor [53]. Conclusions drawn in these publications are often contradictory, and there are limited data on the effects of microbial biostimulants on root crop growth and yield quality.

During the last years, several studies showed that microbial biostimulants can improve not just production, but also food quality, through the increase of some nutrients as well as some plant bioactive compounds, which are beneficial to human health [24,54,55]. It has been found that the application of microbial and nonmicrobial plant biostimulants is able to modify plant primary and secondary metabolism [53,56,57] leading to the synthesis and accumulation of antioxidant molecules (i.e., secondary metabolites). The findings of Rahman and colleagues [58] indicate that plant probiotics increased growth and fruit yield and quality of strawberries. Microbial biostimulants together with mineral fertilization N 105 improved the main quality traits of sugar beet [19]. This coincides with the data of our study which revealed that plant probiotic microorganisms ProbioHumus and NaturGel influenced carrot yield quality under field conditions in nonorganic farms. This was confirmed by the results of the DPPH assay demonstrated that ProbioHumus and NaturGel applied in couple increased antioxidant activity of carrots by 10% and 20% from organic and nonorganic fields, respectively, as compared to untreated ones.

One of the main indicators of carrot quality is the carotenoid content [59,60]. It was shown that organic fertilizers can increase the content of nutrients such as β-carotenes [20]. Concerning the implications of microbial plant biostimulants on improving product quality, Chandrasekaran with coauthors [61] reported that the inoculation of PGPR strain, *Bacillus subtilis* CBR05 induced a significant increase in tomato quality in terms of carotenoids profile. In our study, the highest amount of these pigments was detected in carrots (0.22 mg/g FW) grown in a nonorganic farm and couple applicated with ProbioHumus + NaturGel. The preparations tested on an organic farm did not show a significant effect on the carotenoid accumulation in carrot roots as compared to control.

Some studies have shown that carrots grown with organic fertilizers have a better sweetness than those receiving chemical fertilizers [28,29]. According to our results, quantitative analysis of monosaccharides showed that the tested preparations promoted the formation of sugars in carrot roots—its content increased by 18% as compared to the control. The highest monosaccharide content was found in carrots grown on an organic farm and exposed to ProbioHumus + NaturGel.

According to the literature data carrots contain relatively low amounts of ascorbic acid, but due to its high bioactivity, it is considered to be a significant indicator of the nutritional value of its roots. Considering the high level of raw carrot consumption, carrots can provide an important source of ascorbic acid in the consumers’ diet [2,62]. Of course, its amount in roots depends on the cultivar, although the effect of farming decisions cannot be excluded. Here, our research indicated that the level of ascorbic acid increased due to the application of ProbioHumus in organically grown carrots (Table 5, Figure 4). Meanwhile, in carrots grown on nonorganic farms, a significant increase in ascorbic acid was observed after exposure to ProbioHumus in couple with NaturGel. These data are consistent with the data from other authors who suggest that growth stimulants may affect vitamin C accumulation in carrot root tissues [63,64].

Phenolic compounds are secondary plant metabolites which constitute one of the most widely distributed groups of natural products in plants and are important for the human diet [65]. The growing trend for interest in carrots is attributed to the high content of beneficial phenolic compounds because carrots are among the richest vegetable sources of phenolic acids [66]. Phenol concentrations in carrots were found to range from 50 to 75 mg/100 g fresh weight [26,67]. Our analysis showed that ProbioHumus + NaturGel increased the total phenol accumulation by 10% in carrots from an organic farm. Meanwhile, ProbioHumus rose the content of phenolic compounds to 15% in carrots grown on the nonorganic farm (Table 6).

The carrots are prone to the accumulation of nitrates. The average nitrate content in conventionally grown carrots was showed to be 100–270 mg/kg FW [2,68]. Many authors have indicated that the most important factors affecting the content of nitrates in the carrot are cultivar, environmental conditions and cultivation methods [49,69]. The impact of biostimulants on nitrate content in carrots is ambiguous. Nonmicrobial biostimulants of different nature have been reported to reduce nitrate levels in carrot roots [64,70]. On the other hand, there are studies that the biostimulant application did not affect the nitrate content in carrot roots [25]. In our study regardless of the biostimulant used, organic carrots contain about 2-fold lower content of nitrate than nonorganic ones, and probiotic preparations did not show a significant effect on nitrate accumulation (Figure 6).

In conclusion, microbial biostimulant ProbioHumus positively affected carrot growth and the formation of productivity elements. ProbioHumus and NaturGel applied in couple promoted the antioxidant activity and accumulation of carotenoids, monosaccharides, ascorbic acid, and phenols in carrot roots cultivated on organic and nonorganic farms. Probiotic preparations induced a slight decrease in nitrate accumulation in carrot roots from both farms. Thus, the use of probiotic microorganisms can be the potential alternative for chemical fertilizers in crop production and can help to avoid harmful impacts on the quality of vegetables. Our study suggests that organic carrots yield quality can exceed conventional management in yield quality. Furthermore, the integrated nutrient management that uses microbial preparations in complex with chemical fertilizers helps us to solve the agroenvironmental problems reducing the use of fertilizers. Microbial biostimulants ProbioHumus and NaturGel were effective at low doses, thus can be recommended as an element of cultivation for creating environmentally friendly technologies. In-depth studies into the effects of microbial biostimulants on the growth and development of crops and yield quality will expand the knowledge of responses between root crops and microbes and provide farmers with the tools necessary for sustainable agriculture.

## Figures and Tables

**Table 1:** Effect of probiotic preparations on morphometric parameters of carrot root cultivated under controlled laboratory conditions.

Treatment (1 mL/100 mL)	Average Mass (g)		Average Length (cm)	Average Width (cm)
	Fresh	Dry		
Control (H_2_O)	3.64 ± 0.28 a	0.31 ± 0.02 a	3.19 ± 0.29 a	1.31 ± 0.10 a
NaturGel	3.52 ± 0.31 a	0.33 ± 0.03 a	3.19 ± 0.30 a	1.29 ± 0.12 a
ProbioHumus	3.68 ± 0.20 a	0.35 ± 0.04 a	3.91 ± 0.40 b	1.41 ± 0.11 b
ProbioHumus + NaturGel	4.12 ± 0.35 b	0.40 ± 0.02 b	4.40 ± 0.41 bc	1.72 ± 0.14 bc
**Treatment (2 mL/100 mL)**				
NaturGel	3.73 ± 0.29 a	0.32 ± 0.02 a	4.59 ± 0.41 b	1.43 ± 0.11 b
ProbioHumus	4.02 ± 0.22 b	0.43 ± 0.02 b	5.75 ± 0.48 bc	1.82 ± 0.20 c
ProbioHumus + Naturgel	4.92 ± 0.43 c	0.51 ± 0.04 bc	6.32 ± 0.51 c	2.12 ± 0.18 c
**Treatment (4 mL/100 mL)**				
NaturGel	3.61 ± 0.40 a	0.31 ± 0.03 a	4.12 ± 0.42 b	1.38 ±0.15 b
ProbioHumus	4.12 ± 0.33 b	0.44 ± 0.05 b	5.80 ± 0.60 bc	1.85 ± 0.20 c
ProbioHumus + Naturgel	4.83 ± 0.43 bc	0.48 ± 0.04 bc	6.41 ± 0.58 c	2.01 ± 0.16 c

Values reported are mean of thirty root crops with standard deviation. Means with different letters in the same column are significantly different (*p* < 0.05).

**Table 2:** Effect of ProbioHumus and NaturGel application on carrot morphometric parameters at small plots.

Treatment	Average Mass (g)		Average Length (cm)	Average Width (cm)
Organic	Fresh	Dry		
Control	100.65 ± 8.7 c	11.32 ± 1.0 c	15.12 ± 1.4 a	2.74 ± 0.21 c
ProbioHumus	94.67 ± 8.5 c	10.54 ± 0.9 c	17.41 ± 2.1 b	2.67 ± 0.28 c
ProbioHumus + NaturGel	112.93 ± 10.3 d	11.75 ± 0.9 c	18.44 ± 1.8 b	2.93 ± 0.31 cd
**Nonorganic**				
Control	125.79 ± 10.2 a	13.01 ± 0.51 a	17.63 ± 1.5 a	3.21 ± 0.22 a
ProbioHumus	136.90 ± 9.8 b	13.89 ± 0.66 b	15.87 ± 1.3 b	3.85 ± 0.31 b
ProbioHumus + Naturgel	136.13 ± 10.1 b	13.80 ± 0.71 b	18.14 ± 1.7 a	3.53 ± 0.31 ab

Values reported are mean of thirty root crops with standard deviation. Means with different letters in the same column are significantly different (*p* < 0.05).

**Table 3:** Effect of ProbioHumus and NaturGel application on carrot morphometric parameters at large plots.

Treatment	Average Mass (g)		Average Length (cm)	Average Width (cm)
Organic	Fresh	Dry		
Control	80.8 ± 2.4 a	8.97 ± 0.03 a	17.1 ± 0.2 bc	2.7 ± 0.1 a
ProbioHumus	97.2 ± 4.8 b	11.08 ± 0.03 b	17.6 ± 0.1 ab.	2.7 ± 0.1 a
ProbioHumus + NaturGel	88.5 ± 3.5 ab	9.66 ± 0.03 ab	18.1 ± 0.3 c	2.8 ± 0.1 a
**Nonorganic**				
Control	95.3 ± 2.5 b	14.01 ± 0.09 c	15.1 ± 0.5 a	2.9 ± 0.1 b
ProbioHumus	115.5 ± 3.8 c	15.60 ± 0.04 d	17.4 ± 0.6 bc	2.9 ± 0.3 b
ProbioHumus + Naturgel	97.5 ± 5.4 b	14.42 ± 0.06 c	18.4 ± 0.3 c	2.6 ± 0.1 a

Values reported are mean of thirty root crops with standard deviation. Means with different letters in the same column are significantly different (*p* < 0.05).

**Table 4:** Effect of ProbioHumus and NaturGel application on monosaccharide concentration in carrot roots.

Treatment	Monosaccharides (mg/g FM)
Organic	
Control	5.13 ± 0.31 a
ProbioHumus	6.08 ± 0.57 ab
ProbioHumus + NaturGel	6.81 ± 0.62 b
**Nonorganic**	
Control	4.83 ± 0.38 a
ProbioHumus	5.76 ± 0.44 ab
ProbioHums + NaturGel	6.07 ± 0.53 b

Values reported are mean of three experimental repeats with standard deviation. Means with different letters are significantly different (*p* < 0.05).

**Table 5:** Effect of ProbioHumus and NaturGel application on ascorbic acid content in carrot roots.

Treatment	Ascorbic Acid (μg/g FM)
Organic	
Control	41.32 ± 0.40 a
ProbioHumus	47.62 ± 0.38 b
ProbioHumus + NaturGel	45.70 ± 0.40 b
**Nonorganic**	
Control	47.41 ± 0.40 b
ProbioHumus	53.59 ± 0.51 bc
ProbioHumus + NaturGel	62.57 ± 0.58 c

Values reported are mean of three experimental repeats with standard deviation. Means with different letters are significantly different (*p* < 0.05).

**Table 6:** Effect of biostimulant application on accumulation of phenolic compounds in carrot roots.

Treatment	Total phenolics (GAE μg/g)
Organic	
Control	87.98 ± 5.0 a
ProbioHumus	82.46 ± 8.1 a
ProbioHumus + NaturGel	97.33 ± 7.1 b
**Nonorganic**	
Control	90.66 ± 5.4 a
ProbioHumus	104.60 ± 7.8 b
ProbioHums + NaturGel	88.23 ± 8.1 a

Values reported are mean of three experimental repeats with standard deviation. Means with different letters are significantly different (*p* < 0.05).

## Data Availability

The data supporting reported results can be found in the archive of scientific reports of Nature Research Centre.
